# Leptin Protects Against the Development and Expression of Cocaine Addiction-Like Behavior in Heterogeneous Stock Rats

**DOI:** 10.3389/fnbeh.2022.832899

**Published:** 2022-03-03

**Authors:** Lieselot L. G. Carrette, Cristina Corral, Brent Boomhower, Molly Brennan, Caitlin Crook, Clara Ortez, Kokila Shankar, Sierra Simpson, Lisa Maturin, Leah C. Solberg Woods, Abraham A. Palmer, Giordano de Guglielmo, Olivier George

**Affiliations:** ^1^Department of Psychiatry, University of California, San Diego, La Jolla, CA, United States; ^2^Department of Neuroscience, The Scripps Research Institute, La Jolla, CA, United States; ^3^Department of Internal Medicine, Section on Molecular Medicine, Wake Forest University School of Medicine, Winston-Salem, NC, United States; ^4^Institute for Genomic Medicine, University of California, San Diego, La Jolla, CA, United States

**Keywords:** psychostimulant, outbred strains, substance-related disorders, hormones, bodyweight

## Abstract

Cocaine affects food intake, metabolism and bodyweight. It has been hypothesized that feeding hormones like leptin play a role in this process. Preclinical studies have shown a mutually inhibitory relationship between leptin and cocaine, with leptin also decreasing the rewarding effects of cocaine intake. But prior studies have used relatively small sample sizes and did not investigate individual differences in genetically heterogeneous populations. Here, we examined whether the role of individual differences in bodyweight and blood leptin level are associated with high or low vulnerability to addiction-like behaviors using data from 306 heterogeneous stock rats given extended access to intravenous self-administration of cocaine and 120 blood samples from 60 of these animals, that were stored in the Cocaine Biobank. Finally, we tested a separate cohort to evaluate the causal effect of exogenous leptin administration on cocaine seeking. Bodyweight was reduced due to cocaine self-administration in males during withdrawal and abstinence, but was increased in females during abstinence. However, bodyweight was not correlated with addiction-like behavior vulnerability. Blood leptin levels after ∼6 weeks of cocaine self-administration did not correlate with addiction-like behaviors, however, baseline blood leptin levels before any access to cocaine negatively predicted addiction-like behaviors 6 weeks later. Finally, leptin administration in a separate cohort of 59 animals reduced cocaine seeking in acute withdrawal and after 7 weeks of protracted abstinence. These results demonstrate that high blood leptin level before access to cocaine may be a protective factor against the development of cocaine addiction-like behavior and that exogenous leptin reduces the motivation to take and seek cocaine. On the other hand, these results also show that blood leptin level and bodyweight changes in current users are not relevant biomarkers for addiction-like behaviors.

## Introduction

Cocaine is used primarily for its pleasurable effects by young adults. Overall, 5.5 million Americans reported using cocaine in 2019, which includes 1 million with cocaine use disorders (Samhsa, 2020). While most research on cocaine focuses on its rewarding effect, cocaine also has effects on food intake (Mitchell and Roseberry, 2019) and metabolism (Billing and Ersche, 2015). These results suggest that cocaine use affects bodyweight control and feeding hormones, such as the satiety hormone leptin (Schwartz et al., 1996; Friedman, 2014). This is of therapeutic interest, as increased weight gain during cocaine abstinence can hinder recovery (Cochrane et al., 1998; Billing and Ersche, 2015; Bruening et al., 2018) and feeding hormones can affect rewarding effects of drugs in addition to food. However, the exact role of cocaine self-administration on bodyweight and leptin level, and the effect of leptin on cocaine-seeking is unclear, partly due to the lack of longitudinal studies and experiments with low sample size or identical inbred animals.

Converging lines of evidence suggest that cocaine may alter leptin levels and that leptin may modulate cocaine addiction-like behaviors. Both cocaine and leptin modulate the dopaminergic system and interact during cocaine and food self-administration (Carroll et al., 1989; Figlewicz et al., 2003; Fulton et al., 2006; Hommel et al., 2006). Cocaine has transient anorexigenic effects (Balopole et al., 1979), followed by a compensatory increase in the consumption of fat and carbohydrates (Bane et al., 1993) associated with increased metabolism (Billing and Ersche, 2015) and reduced leptin levels (You et al., 2019). On the other hand, leptin signaling attenuates cocaine-induced locomotor activity (Lee et al., 2018), decreases cocaine reward in a conditioned place preference test (Shen et al., 2016; You et al., 2016) and accelerates extinction from cocaine seeking (You et al., 2016) in rodents. Similar results of the effect of cocaine on feeding have been found in clinical studies (Castro et al., 1987; Weddington et al., 1990; Ersche et al., 2013), but the relationship with leptin is less clear. Reduced leptin levels were reported for crack cocaine users (Ersche et al., 2013; Levandowski et al., 2013; Escobar et al., 2018), but a controlled cocaine-administration study found no effect on leptin levels (Bouhlal et al., 2017). Moreover, in contrast to the results in rats, increased leptin levels were reported to lead to higher cocaine craving in humans (Martinotti et al., 2017), leaving the role of leptin levels on cocaine-seeking unclear.

To address this gap in the literature, we tested the hypothesis that cocaine self-administration leads to increased bodyweight during abstinence through reduced blood leptin levels that contribute to increased cocaine-seeking. A sub-hypothesis is that leptin may be a protective factor against the development of cocaine addiction-like behavior. To this end, we used an animal model of extended access (12 h per day for ∼3 weeks) to intravenous cocaine self-administration in outbred heterogeneous stock (HS) rats (Hansen and Spuhler, 1984; Baud et al., 2013), which mimic the diversity of the human population (Woods and Mott, 2017). Cocaine intake (fixed ratio), motivation to seek cocaine [progressive ratio (PR)], resistance to punishment (contingent footshock) and withdrawal-induced irritable-like behavior (bottle brush) were evaluated and used to calculate an addiction index, which is a composite *Z*-score of the different addiction-like behaviors that is assigned to each rat as a measure of the addiction-like phenotype. To evaluate the impact of cocaine self-administration on bodyweight and blood leptin levels, we used phenotypic data from 306 animals and 120 blood samples from a subset of 60 of these rats from the Cocaine Biobank (Carrette et al., 2021b). We also used a separate cohort of 59 HS rats to test the causal effect of exogenous leptin administration on cocaine-seeking.

## Materials and Methods

### Animals

Male and female radio-frequency identification (RFID) tagged HS rats (Rat Genome Database NMcwiWFsm:HS #13673907, sometimes referred to as N/NIH:HS) were provided by Dr. Leah Solberg Woods. Rats were shipped at 3–4 weeks of age, quarantined, and then pair-housed on a 12 h/12 h normal light/dark cycle in a temperature (20–22°C) and humidity (45–55%) controlled vivarium with *ad libitum* access to tap water and food pellets (PJ Noyes Company, Lancaster, NH, United States). All procedures were conducted in strict adherence to the National Institutes of Health Guide for the Care and Use of Laboratory Animals and were approved by the Institutional Animal Care and Use Committees of The Scripps Research Institute and UC San Diego. Experiments were performed in cohorts of 48–60 rats. In the first experiment, we used behavioral data from 306 animals from the Cocaine Biobank^1^ (Carrette et al., 2021b) that had survived until the end of the experiment and maintained catheter patency. Next, blood samples from a subset of these animals were selected from the biobank for 30 males (1 of 30 males was excluded as there was insufficient baseline blood sample) and 30 females (1 of 30 females was excluded as the animal had lost catheter patency) with variable addiction index at two time points (baseline and withdrawal). Finally, we used a new separate cohort of 30 males and 29 females to test the effect of leptin on cocaine seeking. During the self-administration paradigm, 4 females and 1 male died and 3 females and 1 male lost catheter patency, these animals were excluded from all subsequent leptin testing. During abstinence in the homecage, another 5 females and 4 males died, and 1 female and 5 males lost catheter patency, these animals were excluded from the extinction testing with leptin. For homecage feeding/plasma collection during abstinence, the animals that died were excluded, but not the ones that lost catheter patency during abstinence. One more male died before final plasma leptin collection. At the end of the experiments (18 weeks after catheter surgery), 24 rats either died of either unknown causes or had failed catheter patency testing using a short-acting anesthetic (brevital), leaving 18 males and 16 females.

### Drugs

Cocaine HCl (National Institute on Drug Abuse, Bethesda, MD, United States) was dissolved in 0.9% sterile saline and administered intravenously at a dose of 0.5 mg/kg/0.1 ml per infusion. Leptin (R&D systems, Minneapolis, MN, United States) was dissolved in 0.9% sterile saline and administered intravenously at a dose of 0.6 mg/kg/infusion.

### Timeline

**FIGURE 1:**
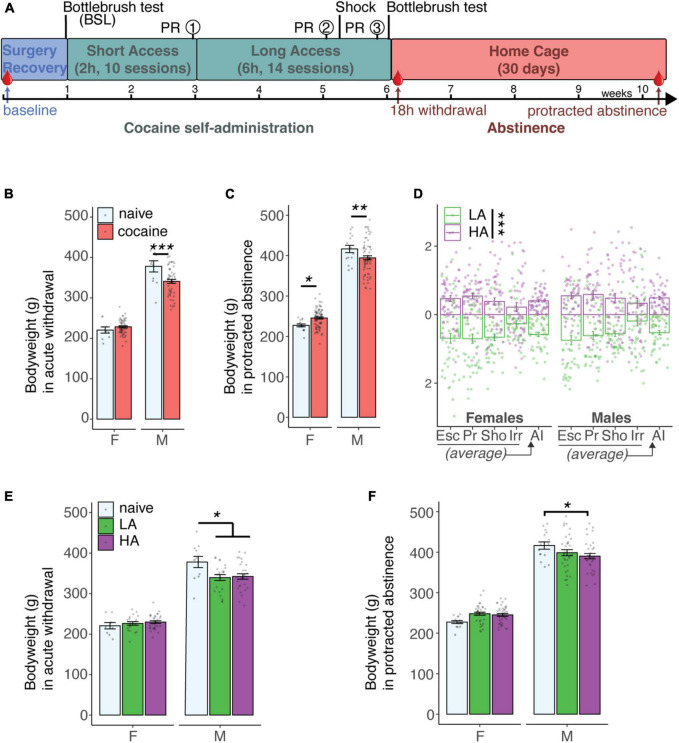
Bodyweights rats. **(A)** Cocaine self-administration behavioral timeline. The red droplet indicates blood collection timepoints. **(B)** Body weight per sex in acute withdrawal following cocaine self-administration compared to naive littermates shows a significant difference between males with ***p = 0.00025 [two-way ANOVA with sex and group as between variables: Group × Sex *F*(1,120) = 9.62 with *p* = 0.0024, main effects of Group *F*(1,120) = 10.67 with *p* = 0.0014 and Sex *F*(1,120) = 507.71 with *p* < 0.0001] N: F = 9 naive + 53 cocaine, *M* = 11 naive + 51 cocaine; 3M + 1F cocaine were removed as outliers. **(C)** Body weight per sex in protracted abstinence following cocaine self-administration compared to naive littermates shows a significant difference between males with **p = 0.018 and females *p = 0.045 [two-way ANOVA with sex and group as between variables: Group × Sex *F*(1,170) = 9.75 with *p* = 0.0021 and main effects of Sex *F*(1,170) = 507.71 with *p* < 0.0001] N: F = 15 naive + 76 cocaine, *M* = 15 naive + 68 cocaine; 1M naive + 3M cocaine were removed as outliers. **(D)** Individual escalation (Esc), motivation (Pr), compulsivity (Sho) *Z*-scores and addiction index show the distinction of the animals with high (HA) and low (LA) addiction-like behaviors. Bonferroni corrected pairwise *t*-tests were significant (***p < 0.0001) for each score in males and females. N: F = 74 HA + 56 LA, *M* = 65 HA + 60 LA. **(E)** Body weight per sex further split per group in acute withdrawal following cocaine self-administration showed a similar significant reduction in bodyweight for both HA (*p = 0.027) and LA (*p = 0.020) males compared to naive males [two-way ANOVA with sex and group as between variables: Group × Sex *F*(2,118) = 4.69 with *p* = 0.011] N: F = 9 naive + 20 LA + 33 HA, *M* = 11 naive + 23 LA + 28 HA; 1F + 3M cocaine were removed as outliers. **(F)** Body weight per sex further split per group in protracted abstinence following cocaine self-administration showed a significant reduction in bodyweight for the HA males (*p = 0.039) compared to naive males [two-way ANOVA with sex and group as between variables: Group × Sex *F*(2,167) = 5.35 with *p* = 0.0056] N: F = 15 naive + 35 LA + 40 HA, *M* = 15 naive + 34 LA + 34 HA; 1M naive, 1F + 3M cocaine were removed as outliers.

The timeline of the experiment is illustrated in Figure 1A. Animals were implanted with an intravenous (*i.v.*) catheter and given access to cocaine self-administration for 10 short access (ShA) and 14 long access (LgA) sessions. Withdrawal was tested by comparing irritability-like behavior at baseline and 18 h into withdrawal using the bottle-brush test. Compulsive-like behavior was tested using 1 session with contingent (30%) footshock as an aversive consequence to lever pressing. The motivation for cocaine was tested using a PR schedule of reinforcement after ShA, LgA, and shock sessions. This phenotyping protocol has been described in more detail previously (Carrette et al., 2021b).

### Jugular Vein Catheterization

This surgery inserts a catheter into the jugular vein to allow for intravenous cocaine self-administration. Rats were anesthetized with vaporized isoflurane (1–5%). Intravenous catheters were aseptically inserted into the right jugular vein using the procedure. Catheters consisted of micro-renathane tubing (18 cm, 0.023-inch inner diameter, 0.037-inch outer diameter; Braintree Scientific, Braintree, MA, United States) attached to a 90-degree angle bend guide cannula (Plastics One, Roanoke, VA, United States), embedded in dental acrylic, and anchored with mesh (1 mm thick, 2 cm diameter). Tubing was inserted into the vein following a needle puncture (22G) and secured with a suture. The guide cannula was punctured through a small incision on the back. The outer part of the cannula was closed off with a plastic seal and metal cover cap, which allowed for sterility and protection of the catheter base. Flunixin (2.5 mg/kg, *s.c.*) was administered as analgesic, and cefazolin (330 mg/kg, *i.m.*) as antibiotic. Rats were allowed 5 days for recovery prior to self-administration. They were monitored and flushed daily with heparinized saline (10 U/ml of heparin sodium; American Pharmaceutical Partners, Schaumberg, IL, United States) in 0.9% bacteriostatic sodium chloride (Hospira, Lake Forest, IL, United States) that contained 52.4 mg/0.2 ml of cefazolin. Catheter patency was tested throughout and at the end of LgA sessions using a short-acting anesthetic (brevital), any animal that failed the test was excluded from the study.

### Behavioral Phenotyping

The rats were subjected to only one behavioral procedure per day, during their dark cycle. Detailed protocols can be found in the George lab protocol repository on protocols.io.^2^

*- Operant self-administration.* The rats were allowed to self-administer cocaine individually in operant chambers (29 cm × 24 cm × 19.5 cm; Med Associates, St. Albans, VT, United States) that were enclosed in lit, sound-attenuating, ventilated environmental cubicles and computer-controlled. Each chamber was equipped with two retractable levers that would only be extended for the duration of the test. A press on the right lever activated an infusion pump on a fixed ratio (FR1) schedule to deliver the drug infusion through plastic catheter tubing over 5 s, followed by a 20 time out during which pressing the lever had no scheduled consequences. Right lever presses also activated a cue light that remained illuminated during the time-out. Pressing the left, inactive lever had no scheduled consequences. The rats went through 10 ShA sessions of 2 h and 14 LgA sessions of 6 h. Five sessions were performed per week, with a break over the weekend.

- *Progressive Ratio (PR) responding* was tested to assess motivation. Number of lever presses required to receive a drug infusion increase progressively (according to the following schedule: 1, 2, 4, 6, 9, 12, 15, 20, 25, 32, 40, 50, 62, 77, 95, 118, 145, 178, …). When the rat did not achieve the required number of presses for the next infusion in the hour following the last infusion, the session was terminated with the breakpoint defined as the last achieved ratio. PR tests were performed after ShA, after LgA, and after foot shock tests. For one cohort, two additional PR tests were performed with intravenous (*i.v.*) leptin treatment (0.6 mg/kg in saline) (You et al., 2016) or saline vehicle, 30 min before the session using a Latin square design.

- *Compulsive-like responding using contingent foot shock.* In this self-administration session, 30% of active lever presses were randomly associated with a foot shock (0.3 mA for 0.5 s), delivered through the floor grid.

- *Withdrawal-induced irritable-like behavior:* Rats were placed in the back of a clean cage, irritated by rotating a bottlebrush near their whiskers for 5 s followed by rotating and slowly withdrawing for 5 s, whilst being scored for defensive (escape, jumping, vocalization, grooming, digging) and aggressive (exploration, boxing, biting, following, latency biting) irritable-like behavior. The test was repeated 10 times per animal, with 10 s intervals and scored by 3 observers. The test was performed before ShA to cocaine as a measurement of baseline behavior and 18 h in withdrawal following LgA to cocaine.

- *Extinction responding.* The subset of animals that were tested for cocaine-seeking with leptin treatment were, after 7 weeks of forced abstinence, re-introduced in the SA chambers for 2 h with access to the levers. The right lever press still activated a cue light, but did not result in drug delivery. Three sessions were performed, separated by 1 week of abstinence. Session 1 and session 3 animals received *i.v.* saline, session 2 animals received *i.v.* leptin (0.6 mg/kg in saline), 30 min before the start of the session.

### Blood Collection and Analysis

*- Baseline blood (before cocaine):* Retroorbital bleeds were performed under anesthesia with the eye numbed by a topical ophthalmic anesthetic (proparacaine hydrochloride).

*- Terminal (acute cocaine withdrawal: euthanized 18 h in withdrawal after a LgA session or protracted cocaine abstinence: euthanized*∼*3–4 weeks after the last cocaine intake during SA sessions*)*:* Blood was collected through cardiac puncture after CO_2_ inhalation.

Fresh blood was collected in EDTA coated tubes to avoid coagulation and was centrifuged at 2,000 g at room temperature (RT) for 10 min. The supernatant plasma was immediately transferred per 500 μl aliquots into fresh tubes, scored for quality on a scale from 0 to 6, snap-frozen on dry ice, and stored at –80°C. Frozen aliquots were thawed on ice and analyzed using a Leptin Rat ELISA kit (Invitrogen, Waltham, MA, United States).

### Data Analysis

**FIGURE 2:**
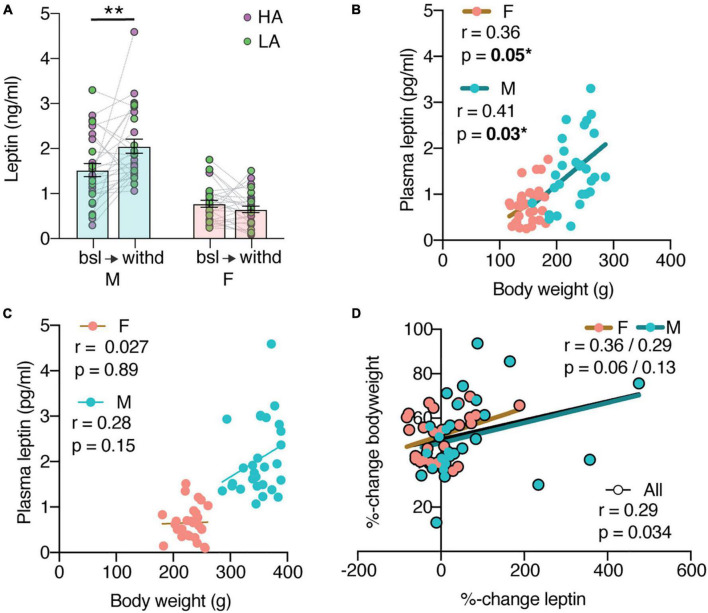
Plasma leptin levels before and after cocaine self-administration. **(A)** Male leptin levels significantly increase from baseline (bsl) to withdrawal (withd) (**p = 0.0012) [mixed effects analysis with sex as between and time as within variables: Timepoint × Sex *F*(1,52) = 10.33 with *p* = 0.0022, main effect of sex *F*(1,56) = 62.64 with *p* < 0.0001]. N: F = 29 bsl + 27 withd, *M* = 28 bsl + 28 withd; 1M bsl, 2F + 1M withd values were excluded as outliers. **(B)** Baseline leptin levels positively correlate to bodyweight (M: r = 0.41, *p = 0.03; F: r = 0.36, *p = 0.05). **(C)** Withdrawal leptin levels do not correlate to bodyweight (M: r = 0.28, *p* = 0.15; F: r = 0.027, *p* = 0.89). **(D)** Percent-change bodyweight does not correlate with the percent-change leptin (M: r = 0.29, *p* = 0.13; F: r = 0.36, *p* = 0.064).

**FIGURE 3:**
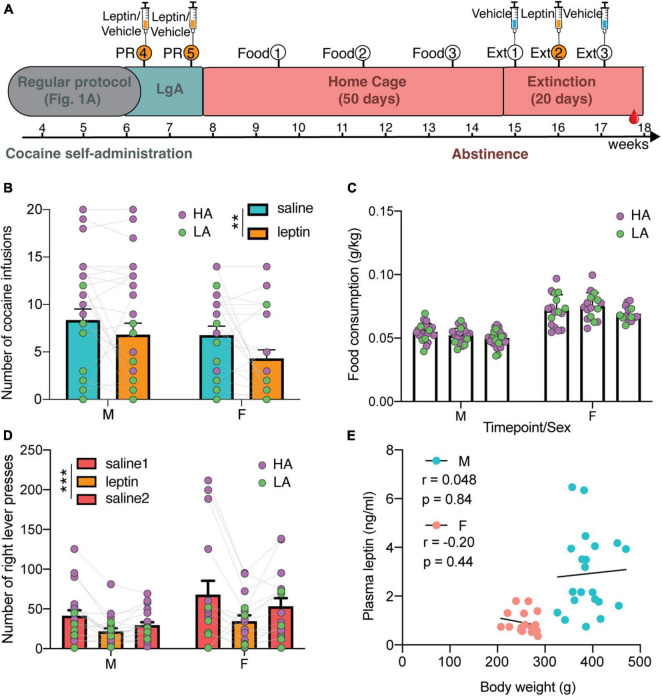
Additional testing in one cohort of animals for leptin and food intake. **(A)** Timeline of additional experiments for characterizing the effect of exogenous leptin on cocaine seeking (syringes indicate i.v. treatment with leptin and/or vehicle), and the effect of cocaine abstinence on food intake and weight. At the end of LgA in the protocol as depicted in Figure 1A, 2 PR sessions were added, separated by a LgA session. During a 7-week abstinence, food intake (Food) was measured every 2 weeks. After 7 weeks, animals were returned to the self-administration chambers for three extinction sessions (Ext), each separated by a week. **(B)** Both males and females significantly reduced their cocaine intake after leptin treatment [two-way ANOVA: Treatment × Sex *F*(1,48) = 0.46 with *p* = 0.50, Treatment *F*(1,48) = 8.64 with **p = 0.0051, and Sex *F*(1,48) = 1.92 with *p* = 0.17] N: F = 22, *M* = 28. **(C)** Females had a higher weight-normalized food intake than males, but there was no change over time [mixed effects analysis with time as within and sex as between variables: Timepoint × Sex *F*(2,70) = 1.42 with *p* = 0.25, simple main effect of Sex *F*(1,39) = 139.7 with *p* < 0.0001 and Timepoint *F*(1.786,62.52) = 3.09 with *p* = 0.058] N: F = 17, *M* = 24; 3M at time 1, 1F and 2M at time 2, and 2F at time 3 were removed as outliers. **(D)** Both males and females pressed significantly less during extinction after leptin treatment [mixed two-way ANOVA with treatment as within and sex as between variables: Treatment × Sex *F*(2,66) = 0.89 with *p* = 0.42 and simple main effect of Treatment *F*(1.429, 47.14) = 11.69 with ***p = 0.0004 and Sex *F*(1,33) = 3.30 with *p* = 0.078] N: F = 16, *M* = 19. **(E)** Abstinence leptin levels do not correlate to bodyweight (M: r = 0.048, *p* = 0.84; F: r = –0.20, *p* = 0.44) N: F = 17, *M* = 23; 2M were removed as outlier for weight and/or leptin.

**FIGURE 4:**
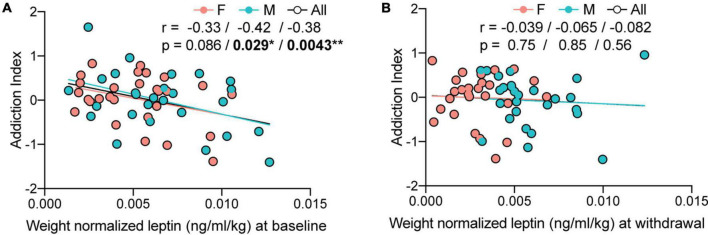
Plasma leptin analysis at baseline. **(A)** The addiction index negatively correlates with weight-normalized baseline leptin levels (M: r = –0.42, *p = 0.029; F: r = –0.33, *p* = 0.086; all: *r* = –0.38, **p = 0.0043), N: 28F + 27M; 1M value was excluded as outlier, 1F and 1M were excluded for missing irritability data. **(B)** The addiction index does not correlate with weight-normalized leptin levels in withdrawal (M: r = –0.065 *p* = 0.85; F: r = –0.039, *p* = 0.75; all: *r* = –0.082, *p* = 0.56), N: 26F + 27M; 2F + 1M values were excluded as outliers, 1F and 1M were excluded for missing irritability data.

Data were analyzed with RStudio, using the rstatix package (for aggregated cohorts in Figure 1) and Graphpad Prism (for single cohorts in Figures 2–4). Values above the third quartile (Q3) + 1.5× interquartile range (IQR) or below Q1 - 1.5× IQR were considered as outliers and removed from the data. Statistical tests were two-way ANOVA with or without repeated measures as needed and described. For the leptin level change (Figure 2A) and food intake analysis (Figure 3C), data values were missing at different timepoints due to outliers, so a mixed-effects model was applied instead of ANOVA, using the maximum likelihood-method. Pairwise differences following a significant main effect were calculated with Bonferroni correction. Weight-normalization was performed as indicated by dividing the measurement (plasma leptin level or food intake) by the individual bodyweight at the same timepoint. An addiction index for each rat was calculated as described before (Carrette et al., 2021b) using the average of the *Z*-scores [(individual value-group average)/group standard deviation] per cohort and per sex for intake, motivation, compulsivity, and irritability as illustrated in Figure 1D. A daily escalation index (E_*i*_) can be calculated for each session i [E_*i*_ = (rewards in LgA session i – average rewards in LgA session 1)/rewards standard deviation in LgA session 1]. The overall escalation index (Esc) was obtained by taking the *Z*-score of the average daily escalation indexes for the last 3 days. The motivation index (Pr) was obtained by taking the *Z*-score of the breakpoint after LgA. The compulsivity index (Sho) was obtained by taking the *Z*-score of the number of rewards obtained in the shock session. The irritability index (Irr) was obtained by taking the *Z*-score of the difference in aggressive score in withdrawal after LgA compared to baseline. The addiction index was initially calculated using the average of the escalation, motivation, compulsivity, and irritability indices. However, because the irritability-like behavior was missing for some of the animals and given that we found no correlation between the irritability index and the escalation, motivation, and compulsivity indices we also calculated an addiction index without the irritability index (Figures 1–3). Overall, the results obtained with or without the irritability index were similar (see section “Results”).

## Results

### Cocaine Self-Administration Alters Bodyweight in a Sex-Dependent Manner

Bodyweights at the end of the cocaine self-administration protocol (Figure 1A) were found to be significantly different compared to naive littermates in a sex dependent manner, both during acute withdrawal [Figure 1B, two-way ANOVA with sex and group as between variables has a significant two-way interaction of Group × Sex *F*(1,120) = 9.62 with *p* = 0.0024, and significant main effects of Group *F*(1,120) = 10.67 with *p* = 0.0014 and Sex *F*(1,120) = 507.71 with *p* < 0.0001; N: F = 9 naive + 53 cocaine, *M* = 11 naive + 51 cocaine; 3M + 1F cocaine were removed as outliers] and protracted abstinence [Figure 1C, two-way ANOVA with sex and group as between variables: Group × Sex *F*(1,170) = 9.75 with *p* = 0.0021 and main effect of Sex *F*(1,170) = 507.71 with *p* < 0.0001; N: F = 15 naive + 76 cocaine, *M* = 15 naive + 68 cocaine; 1M naive + 3M cocaine were removed as outliers]. Analysis of simple main effects for group level was performed with a Bonferroni adjustment. There was a statistically significant difference in mean bodyweight for both males in withdrawal [*F*(1, 120) = 14.2, *p* = 0.00025], males in abstinence [*F*(1, 170) = 5.75, *p* = 0.018], whose bodyweight decreased compared to naive males, and females in abstinence [*F*(1,170) = 4.07, *p* = 0.045], whose bodyweight increased compared to naive females. The addiction-like behaviors of these animals have been characterized through the self-administration protocol and can be described by the calculated *Z*-scores of responding during the FR, PR, and shock sessions, or averaged in an overall addiction index (Figure 1D). Animals with a positive addiction index were classified as having high addiction-like behaviors (HA), those with a negative addiction index as having low addiction-like behaviors (LA). Bonferroni-corrected pairwise tests for each score in males and females showed significant differences between HA and LA animals (*p* < 0.0001, N: F = 74 HA + 56 LA, *M* = 65 HA + 60 LA). When looking at the bodyweight differences split further between naive LA and HA groups, the sex difference obviously remains during acute withdrawal [Figure 1E, two-way ANOVA with sex and group as between variables: Group × Sex *F*(2,118) = 4.69 with *p* = 0.011; N: F = 9 naive + 20 LA + 33 HA, *M* = 11 naive + 23 LA + 28 HA; 1F + 3M cocaine were removed as outliers] and protracted abstinence [Figure 1F, two-way ANOVA with sex and group as between variables: Group × Sex *F*(2,167) = 5.35 with *p* = 0.0056; N: F = 15 naive + 35 LA + 40 HA, *M* = 15 naive + 34 LA + 34 HA; 1M naive, 1F + 3M cocaine were removed as outliers]. As above, there was a statistically significant difference in mean bodyweight for both males in withdrawal [*F*(2, 118) = 7.07, *p* = 0.001] and males in abstinence [*F*(1, 170) = 5.75, *p* = 0.018], whose bodyweight decreased after cocaine intake. But no significant main effect was seen for females in abstinence [*F*(2,167) = 2.33, *p* = 0.10]. Bonferroni-corrected pairwise comparisons between the male subgroups showed a similar significant reduction in bodyweight compared to naive males for both HA (*p* = 0.027) and LA (*p* = 0.020) groups in withdrawal and between naive males and the HA group (*p* = 0.039) in abstinence, with a similar trend in the LA group. Note that the results of this analysis are unchanged when using an addiction index that includes irritability-like behavior. Cocaine males also weigh significantly less than naive males in withdrawal [*F*(1, 120) = 14.2, *p* = 0.00025] and abstinence [*F*(1, 125) = 7.92, *p* = 0.006]. Cocaine females have a trend to weigh significantly more than naive females in abstinence, but this did not reach significance with the reduced animal number for which irritability-like behavior was available [*F*(1, 125) = 2.18, *p* = 0.143]. In addition, there were no significant differences between HA and LA males or females in abstinence or withdrawal. Overall, these results indicate that a history of cocaine self-administration differently affects bodyweight in male (decrease) vs. female (increase) but that these effects do not depend on the severity of addiction-like behaviors (HA vs. LA).

### Cocaine Self-Administration Increases Endogenous Leptin Levels in Males but Not Females

Since it is documented that cocaine influences leptin, we suspected leptin of being part of the mechanism through which cocaine asserts its effect on bodyweight. We next looked at endogenous leptin levels, within subject, before (as a proxy of naive animals) and after cocaine self-administration. Blood samples were selected from the Cocaine Biobank (Carrette et al., 2021b) from 60 animals, half male and half female. The withdrawal timepoint was chosen as it gave the most significant change in bodyweights. The leptin levels were significantly different between males and females (Figure 2A) [main effect of sex *F*(1,56) = 62.64 with *p* < 0.0001; N: F = 29 bsl + 27 withd, *M* = 28 bsl + 28 withd; 1M bsl, 2F + 1M withd values were excluded as outliers] and changed differently over the cocaine self-administration [mixed effects analysis with sex as between and time as within variables: *F*(1,52) = 10.33 with *p* = 0.0022]. Bonferroni corrected pairwise comparisons showed that male leptin levels significantly increased (*p* = 0.0012). A cocaine-induced leptin level increase could explain the observed decrease in bodyweight. However, cautious interpretation of these results is required as leptin levels are affected by bodyweight and the animals gained weight over the course of the experiment. At baseline, plasma leptin levels correlate positively to the bodyweights (Figure 2B) (M: r = 0.41, *p* = 0.03; F: r = 0.36, *p* = 0.05). This correlation is lost after cocaine self-administration, especially in females (Figure 2C) (M: r = 0.28, *p* = 0.15; F: r = 0.027, *p* = 0.89). Moreover, the percent-change in bodyweight did not correlate with the percent-change in leptin (Figure 2D), (M: r = 0.29, *p* = 0.13; F: r = 0.36, *p* = 0.064). This suggests that a history of cocaine self-administration affects the relationship between leptin and body weight.

### Systemic Leptin Administration Reduces Cocaine Seeking

One cohort of HS animals (*N* = 29 F + 30 M) that completed the cocaine-self administration protocol (Figure 1A) was dedicated to investigate the effect of cocaine on food intake and of leptin on addiction-like behaviors. Specifically the effect of systemic intravenous administration of 0.6 mg/kg leptin, which was shown before to reduce cocaine seeking under extinction conditions (You et al., 2016), was examined on PR responding and reinstatement of cocaine-seeking in a cohort following the earlier described self-administration protocol (Figure 3A). Both male and female rats significantly reduced their cocaine intake in a PR schedule after leptin treatment [Figure 3B, mixed two-way ANOVA with treatment as within and sex as between subject variables: Treatment × Sex *F*(1,48) = 0.46 with *p* = 0.50 and simple main effect of Treatment *F*(1,48) = 8.64 with *p* = 0.0051, N: F = 22, *M* = 28]. There were no sex differences in the test [simple main effect of Sex *F*(1,48) = 1.92 with *p* = 0.17]. Per definition, HA and LA animals respond differently to this test [simple main effect of Group *F*(1,48) = 22.13 with *p* < 0.0001] and both groups were equally affected by leptin treatment [mixed two-way ANOVA with treatment as within and group as between variable has no significant interaction: Treatment × Group *F*(1,48) = 0.11 with *p* = 0.74, and a main simple effect of Treatment *F*(1,48) = 9.37 with *p* = 0.0036]. Following the test, the animals were kept in their home cage for 7 weeks of forced abstinence. Food intake during the dark cycle was measured every 2 weeks during abstinence (Figure 3C). Weight-normalized intake was higher in females [simple main effect of Sex *F*(1,39) = 139.7 with *p* < 0.0001], and was stable over time in both males and females [mixed effects analysis with time as within and sex as between variables: Timepoint × Sex *F*(2,70) = 1.42 with *p* = 0.25, Timepoint *F*(1.786,62.52) = 3.09 with *p* = 0.058; N: F = 17, *M* = 24; 3M at time 1, 1F and 2M at time 2, and 2F at time 3 were removed as outliers]. There was no difference in food intake between HA and LA animals at any of the three timepoints in abstinence [mixed effects analysis: time as within and group as between variables: Timepoint × Group *F*(2,70) = 0.85 with *p* = 0.43 and main effect of Group *F*(1,39) = 0.000027 with *p* = 0.99]. Next, the effect of leptin on cocaine seeking was examined after 7 weeks of abstinence by three extinction sessions, each performed with a one-week interval, over three consecutive weeks. During these sessions the animals were returned to the SA boxes, where they could lever press, but did not receive cocaine. In week 1, cocaine-seeking was tested with vehicle treatment. Week 2 tested the effect of leptin treatment. Finally, vehicle treatment was repeated in week 3 to confirm that the effect was due to leptin and not only due to an extinction of cocaine-seeking. Both male and female rats responded to the treatment with leptin by significantly reducing lever presses compared to vehicle sessions [mixed two-way ANOVA with treatment as within and sex as between variables: Treatment × Sex *F*(2,66) = 0.89 with *p* = 0.42; N: F = 16, *M* = 19], but there was a simple main effect of Treatment [*F*(1.429, 47.14) = 11.69 with *p* = 0.0004] (Figure 3D). Here too, HA and LA animals responded differently to the test [simple main effect of Group *F*(1,33) = 4.85 with *p* = 0.035]. Moreover, there was a different response to the treatment [mixed two-way ANOVA with treatment as within and group as between variable: Treatment × Group *F*(2,66) = 4.97 with *p* = 0.0098], with leptin having a significant effect on HA rats (*post hoc* test *p* < 0.001) but not on LA rats. The latter exhibited significantly less cocaine-seeking than the HA rats. Figure 3E shows that the correlation between blood leptin level and bodyweight was still lost (M: r = 0.048, *p* = 0.84; F: r = –0.20, *p* = 0.44, N: F = 17, *M* = 23; 2M were removed as outlier for weight and/or leptin), and that males exhibited higher endogenous blood leptin levels than females in protracted abstinence [simple main effect of Sex *F*(1,36) = 25.44 with *p* < 0.0001], with levels further increased compared to the levels of the animals at baseline and withdrawal (compare with Figures 2A–C). No significant difference was found between HA and LA [two-way ANOVA with sex and group as between variables: Group × Sex *F*(1,36) = 1.27 with *p* = 0.27 and main effect of Group *F*(1,36) = 1.59 with *p* = 0.22].

### Endogenous Leptin Level at Baseline Protects Against the Development of Addiction-Like Behaviors

We then tested if there was a correlation between endogenous weight-normalized leptin levels and the addiction index. At baseline, weight-normalized leptin levels indeed correlated with the addiction index characterized 6 weeks later (Figure 4A) (M: r = –0.42, *p* = 0.029; F: r = –0.33, *p* = 0.086; all: *r* = –0.38, *p* = 0.0043; N: 28F + 27M; 1M value was excluded as outlier, 1F and 1M were excluded for missing irritability data). There was no significant correlation during withdrawal after 6 weeks of cocaine self-administration (Figure 4B) (M: r = –0.065, *p* = 0.85; F: r = –0.039, *p* = 0.75; all: *r* = –0.082, *p* = 0.56; N: 26F + 27M; 2F + 1M values were excluded as outliers, 1F and 1M were excluded for missing irritability data). The results were the same when using raw plasma leptin levels as demonstrated in the supplementary data (Supplementary Figures 1A,B) and when calculating the addiction index without irritability-like behavior (*r* = –0.30 with *p* = 0.020 at baseline and *r* = –0.17 with *p* = 0.22 in withdrawal). Correlating the weight normalized leptin levels to the different components of the addiction index, the escalation index (Supplementary Figure 1C), motivation index (Supplementary Figure 1D), compulsivity index (Supplementary Figure 1E), and irritability index (Supplementary Figure 1F) shows that the correlation is mostly driven by motivation.

## Discussion

Here we examined the effect of cocaine self-administration on bodyweight and leptin levels, and the effect of leptin on cocaine-seeking in HS rats. Contrary to our working hypothesis, we found reduced bodyweight, and increased leptin in withdrawal and abstinence for males. Plasma leptin levels in withdrawal and abstinence seemed dysregulated by cocaine intake as they lost their typical positive correlation with bodyweight, but no differences between HA vs. LA animals were found. However, when looking at leptin levels in HS rats before the first cocaine self-administration, a negative correlation was found between blood leptin levels and the addiction index 6 weeks later, which indicates that endogenous leptin may be a protective factor against the development of cocaine addiction-like behavior. Finally, administration of leptin decreased cocaine-seeking as measured using a PR schedule of reinforcement during withdrawal and using an extinction session after protracted abstinence. These results demonstrate that leptin provides protective effects against cocaine addiction-like behavior, but that blood leptin levels *per se*, do not predict cocaine-seeking after abstinence.

Leptin is a well-known modulator of bodyweight, through modulation of food intake and food metabolism by acting on the hypothalamic circuitry (Halaas et al., 1995; Farooqi et al., 1999) and by reducing dopamine (DA) signaling in relation to food cues in the mesolimbic circuitry (Hommel et al., 2006; Sadaf Farooqi et al., 2007; Van Der Plasse et al., 2015). Leptin administration, whether systemically or intracranial in the VTA or NAc, has been shown to attenuate cocaine conditioned place preference, cocaine-seeking under extinction conditions and cocaine-induced locomotor activity (You et al., 2016; Lee et al., 2018). In mice, both the injection of the leptin receptor antagonist or deletion of the receptor was shown to enhance cocaine conditioned place preference (Shen et al., 2016). We confirmed and extended these findings by demonstrating that exogenous leptin inhibits cocaine self-administration under a PR schedule in acute withdrawal and under extinction 7 weeks into abstinence. No sex difference was found in the effect of leptin on cocaine-seeking. While leptin reduced PR responding in both HA and LA rats, it reduced cocaine-seeking during extinction only in HA rats, suggesting that HA rats’ cocaine-seeking exhibits a long-lasting sensitivity to leptin.

The effect of cocaine use on blood leptin levels in humans is unclear. Indeed, a history of cocaine use has been shown to increase (Levandowski et al., 2013; Martinotti et al., 2017), decrease (Ersche et al., 2013; Escobar et al., 2018) or have no effect (Bouhlal et al., 2017) on blood leptin levels. In rats, cocaine self-administration and craving causes fluctuation of leptin levels (You et al., 2016, 2019). Both cocaine self-administration and the expectancy of access to cocaine decreases leptin levels, an effect which normalizes during abstinence (You et al., 2016). We did not test the direct effect of cocaine on blood leptin levels, however, we found that after self-administration, blood leptin levels were not correlated with bodyweight anymore, suggesting that a history of cocaine self-administration may dysregulate blood leptin levels and that it may not represent a good biomarker of addiction-like behavior during abstinence. We did find that lower blood leptin levels at baseline were associated with higher addiction-like behaviors 6 weeks later using samples from the Cocaine Biobank. This correlation was mainly driven by the motivational component of the addiction index, which is a similar measure to drug-seeking during reinstatement. The Cocaine Biobank (Carrette et al., 2021b) is a unique resource providing phenotypic data and biological samples from HS rats that underwent state-of-the-art extended access to intravenous cocaine self-administration. As a result of their diverse genotypes, HS rats exhibit diverse addiction-like behavior phenotypes, which mimic the diversity in humans (Woods and Mott, 2017). The negative correlation between blood leptin levels at baseline and addiction-like behavior in the biobank samples confirms our findings that leptin decreases cocaine-seeking and that the inhibitory relationship between leptin and cocaine may be bidirectional, as suggested by You et al. (2016). While the predictive effect observed is small (R^2^∼0.13), it is in line with the protective effect of leptin that we and others observed against cocaine-seeking (You et al., 2016) and reward (Shen et al., 2016; You et al., 2016). The effect of leptin on cocaine-seeking may be related to its inhibitory effect on the brain reward system and the known effect of leptin mutations in mood disorders (Kraus et al., 2001; Atmaca et al., 2002, 2008).

In summary, these results demonstrate that high blood leptin level before access to cocaine may be a protective factor against the development of cocaine addiction-like behavior and that exogenous leptin reduces the motivation to take and seek cocaine. These findings suggest that leptin could be a good target for medication development for the prevention and treatment of cocaine use disorder. However, we show that blood leptin levels fluctuate in withdrawal and abstinence in current users and that it is thus not a relevant biomarker for addiction-like behaviors. Further studies will be required to understand if and how these leptin changes could drive the opposite bodyweight change following cocaine self-administration in males and females.

## Data Availability Statement

The raw data supporting the conclusions of this article will be made available by the authors, without undue reservation.

## Ethics Statement

The animal study was reviewed and approved by Institutional Animal Care and Use Committees of the Scripps Research Institute and University of California, San Diego.

## Author Contributions

LC, GdG, and OG contributed to conception and design of the study. LC, CCr, BB, MB, CO, KS, and SS performed experiments. CCo, BB, and LM organized the database. LS and AP contributed HS rats. LC and OG analyzed the data and wrote the manuscript. All authors contributed to manuscript revision, read, and approved the submitted version.

## Conflict of Interest

The authors declare that the research was conducted in the absence of any commercial or financial relationships that could be construed as a potential conflict of interest.

## Publisher’s Note

All claims expressed in this article are solely those of the authors and do not necessarily represent those of their affiliated organizations, or those of the publisher, the editors and the reviewers. Any product that may be evaluated in this article, or claim that may be made by its manufacturer, is not guaranteed or endorsed by the publisher.

## Footnotes

1
https://www.cocainebiobank.org

2
https://www.protocols.io/workspaces/george-lab
