# Supplementary material for: Leptin Protects Against the Development and Expression of Cocaine Addiction-Like Behavior in Heterogeneous Stock Rats
Source: Front Behav Neurosci. 2022 Mar 3;16:832899. doi: 10.3389/fnbeh.2022.832899 (PMC8934439; doi:10.3389/fnbeh.2022.832899)
Supplement: Supplementary file 1 [file Data_Sheet_1.pdf]

Supplementary Material for:

Leptin protects against the development and expression of cocaine addiction-like behavior in heterogeneous stock rats

L.L.G. Carrette[1], C. Corral[1], B. Boomhower[1], M. Brennan[1], C. Crook[1], C. Ortez[1], K. Shankar[1,2], S. Simpson[1], L. Maturin[1], L.C. Solberg Woods[3], A.A. Palmer[1,4], G. de Guglielmo[1], O. George[1,*]

[1] Department of Psychiatry, UC San Diego, La Jolla, CA, United States.

[2] Department of Neuroscience, The Scripps Research Institute, La Jolla CA, United States.

[3] Department of Internal Medicine, Section on Molecular Medicine, Wake Forest University School of Medicine, Winston-Salem, NC, United States.

[4] Institute for Genomic Medicine, University of California, San Diego, La Jolla, CA, United States.

*olgeorge@health.ucsd.edu

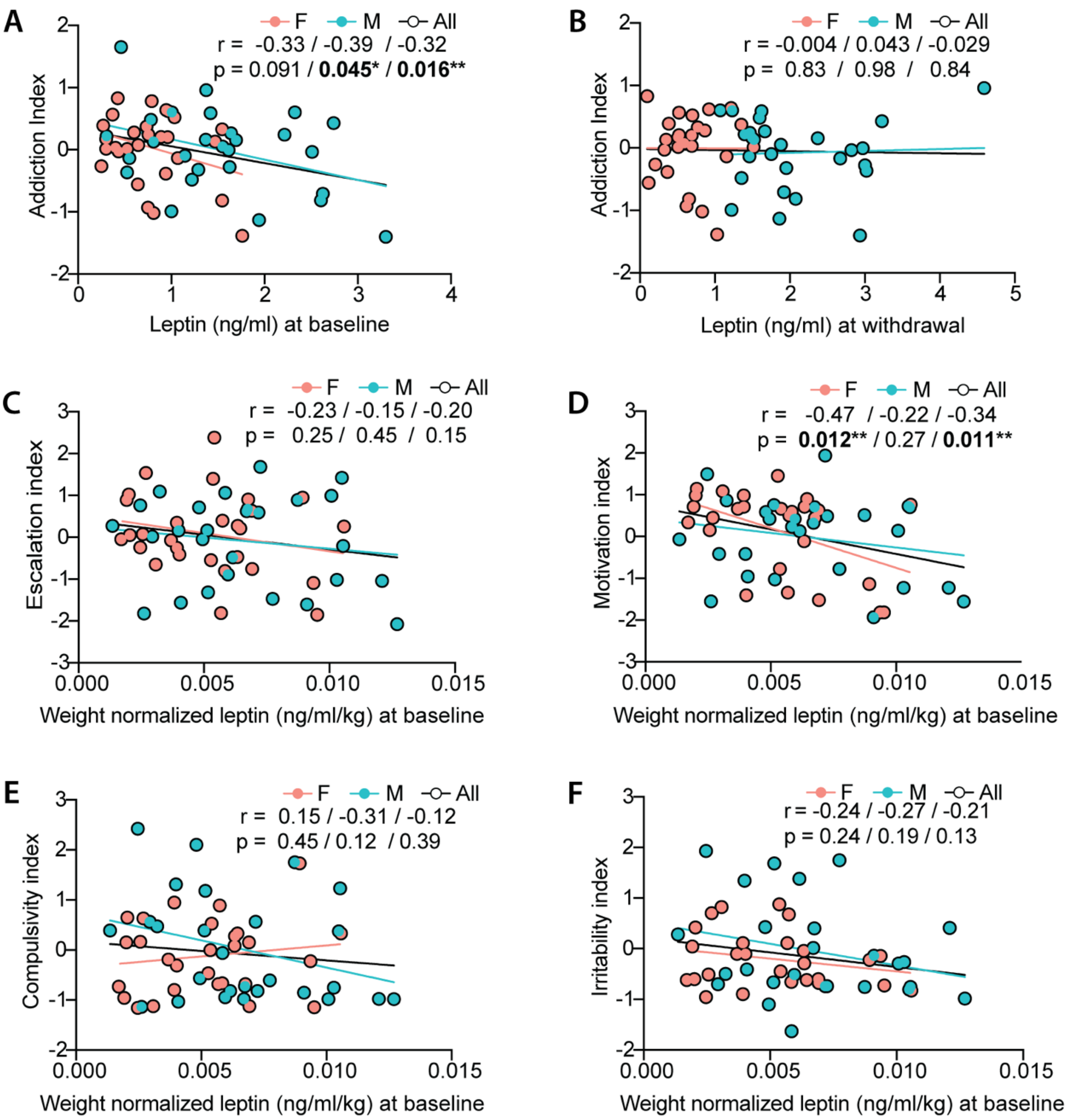

Figure S1. Additional correlations of leptin plasma levels and measures of addiction-like behavior. (A) The addiction index negatively correlates with baseline leptin levels (M: r = -0.39, *p = 0.045; F: r = -0.33, p = 0.091; all: r = -0.32, **p = 0.016), N: 28F+27M; 1M value was excluded as outlier, 1F and 1M were excluded for missing irritability data. **(B)** The addiction index does not correlate with leptin levels in withdrawal (M: r = 0.043, p = 0.98; F: r = -0.004, p = 0.83; all: r = -0.029, p = 0.84), N: 26F + 27M; 2F + 1M values were excluded as outliers, 1F and 1M were excluded for missing irritability data. **(C)** Correlation of weight-normalized baseline leptin levels with the escalation index (based on FR1 responding at the end of LgA) (M: r = -0.15, p = 0.45; F: r = -0.23, p = 0.25; all: r = -0.20, p = 0.15), N: as in A. **(D)** Correlation of weight-normalized baseline leptin levels with the motivation index (based on PR2 responding) (M: r = -0.22, p = 0.27; F: r = -0.47, **p = 0.012; all: r = -0.34, **p = 0.011), N: as in A. **(E)** Correlation of weight-normalized baseline leptin levels with the compulsivity index (based on shock responding) (M: r = -0.31, p = 0.12; F: r = 0.15, p = 0.45; all: r = -0.12, p = 0.39), N: as in A. (**F**) Correlation of weight-normalized baseline leptin levels with the irritability index (based on bottlebrush test) (M: r = -0.27, p = 0.19; F: r = -0.24, p = 0.24; all: r = -0.21, p = 0.13), N: as in A.
